# Evaluation of Process Communication Model training for surgeons and other healthcare professionals: a survey

**DOI:** 10.1111/ans.16351

**Published:** 2021-04-12

**Authors:** Marion I. Andrew, Stephen Wilkinson, Nicole Wylie, Amy Salter

**Affiliations:** ^1^ Department of Anaesthesia Women's and Children's Hospital Adelaide South Australia Australia; ^2^ Department of Surgery Royal Hobart Hospital Hobart Tasmania Australia; ^3^ Department of Paediatric Anaesthesia Women's and Children's Hospital Adelaide South Australia Australia; ^4^ School of Public Health The University of Adelaide Adelaide South Australia Australia

How surgeons communicate, and whether that communication engenders co‐operation, resistance or conflict, is critical to teamwork and patient safety. Surgeons' work environments are complex, stressful, safety–critical and increasingly overlaid by bureaucracy and medico‐legal concerns. Consistently demonstrating excellence in interpersonal skills is challenging. Observational research in theatre teams reveals frequent ‘tension‐filled communicative events’, interpersonal conflict and dysfunctional relationships,^1^ which negatively impact performance, trainee supervision, quality of care and may contribute to burnout.^2^, ^3^, ^4^ Communication is one of the nine surgical competencies of the Royal Australasian College of Surgeons (RACS) relevant to all areas of surgical practice.^5^ RACS recognized a gap in interpersonal and self‐management skills, and since 2010, has hosted within its continuing professional development programme, a course known as Process Communication Model (PCM).

PCM provides a logical framework to understand personality structure and teaches a skillset to adapt communication styles under normal and ‘stressed’ conditions. The model explains how people view the world and express their personality through sub‐conscious patterns of words and behaviour. PCM's basic tenet is that personality is multifaceted and comprises a mix of six personality types, with one being typically dominant and associated with observable speech patterns and body language. Participants learn pattern recognition to identify the preferred communication style of the receiver, and adaptive skills to connect and avoid miscommunication. PCM shares some features with other personality models, for example, DiSC model, Social Style Model and Myers–Briggs Type Indicator.^6^ Unique points of difference include PCM's structured approach to understanding stress and distress behaviour (including unhelpful aggressive and submissive behaviours), targeted skills to prevent and resolve conflict and powerful individual insights into strategies for self‐management.^7^

While immediate PCM course feedback is consistently positive, the ultimate aim of surgical education is translation of knowledge and skills into long‐term practice. We wanted to answer the questions: What are participants' attitudes to the ongoing impact of PCM training in professional and personal life and were they still using the skills? A survey designed using Kirkpatricks' four levels of evaluation (reactions, learning, behaviour and outcomes) was emailed to 769 healthcare professionals who attended PCM courses between February 2010 and October 2017.^8^ In addition to demographic data, 14 questions, scored on a visual analogue scale 0–100 (where 0 = not at all and 100 = extremely/very frequently) addressed professional relevance, skills utility, conflict management, professional and personal relationships, self‐efficacy, stress and well‐being. Free text comments were invited. Ethics approval was obtained.

The response rate was 40% (311 responses). Seventy‐five per cent had attended PCM training between 6 months and 3 years prior, and 25% attended between 3 and 6 years prior. Most (68%) were medical doctors and four major specialty subgroups (surgeons, anaesthetists, medical scientists and administrators/educators) were identified.

Key survey findings in surgeons indicated PCM was relevant (median 80, interquartile range (IQR) 73–90) and recommended (median 99, IQR 85–100) with moderate ease of integration into professional life (median 60, IQR 40–80). Surgeons were still using their pattern recognition skills (median 70, IQR 47–90) and conflict management skills (median 67, IQR 50–78) particularly with colleagues (94%). Evaluation outcomes showed positive impacts on professional (median 80, IQR 57–93) and personal relationships (median 75, IQR 60–92), self‐efficacy (median 75, IQR 54–90), well‐being (median 71, IQR 60–92) and stress reduction (median 60, IQR 47–78). Free text comments were 86% positive indicating personal insights, while 14.0% experienced some challenges in implementing skills. Surgeons' scores were comparable with other subgroups.

Surgeons' reactions demonstrate PCM was valued and relevant in long‐term practice. Skills integration scores and comments reflected the challenges of changing communication habits. Learning and behaviour were analysed together, following the convention that no learning can be said to have occurred, unless there is behavioural change.^8^ The frequently reported use of skills in professional and personal contexts implies that behavioural change occurred. Results suggest surgeons with PCM skills may be better equipped to communicate effectively, may better deal with stress encountered in professional and personal life and may be at less risk of litigation.^9^ Moderate reductions in stress may reflect gradual development of intuitive use. Comparable median scores in the subgroups point to the applicability of PCM to different settings.

PCM training focuses on the rhetorical concepts of communication, and this survey advances our understanding of ‘*how*’ might we prepare surgeons to communicate effectively and manage stressful situations with emotional balance. Study limitations include a course enrolment self‐selection bias for professionals more likely to have an interest in communication, non‐response bias, low response rate bias and varied time periods since respondents' training. Despite these limitations, this survey obtained a body of descriptive data indicating that PCM training offers a learnable framework and durable skillset supporting RACS training standards for communication competency and positive benefits for professional and personal life. Future research plans include prospective long‐term evaluation.

## Author Contributions

**Marion Andrew:** Conceptualization; data curation; formal analysis; methodology; project administration; supervision; writing‐original draft; writing‐review and editing. **Stephen Wilkinson:** Project administration; writing‐review and editing. **Nicole Wylie:** Formal analysis; writing‐original draft; writing‐review and editing. **Amy Salter:** Conceptualization; data curation; formal analysis; writing‐review and editing.

## Conflicts of interest

MIA and SW are certified pro‐bono facilitators of PCM. They necessarily hold a license with Kahler Communications Oceania Ltd to teach PCM. The remaining two authors and acknowledged colleagues had no training in PCM.
